# Retraction: Teixeira et al. RADseq Data Suggest Occasional Hybridization between *Microcebus murinus* and *M. ravelobensis* in Northwestern Madagascar. *Genes* 2022, *13*, 913

**DOI:** 10.3390/genes13112146

**Published:** 2022-11-18

**Authors:** Helena Teixeira, Tobias van Elst, Malcolm S. Ramsay, Romule Rakotondravony, Jordi Salmona, Anne D. Yoder, Ute Radespiel

**Affiliations:** 1 Institute of Zoology, University of Veterinary Medicine Hannover, Bünteweg 17, 30559 Hannover, Germany; 2 Department of Anthropology, University of Toronto, 19 Russell St., Toronto, ON M5S 2S2, Canada; 3 Ecole Doctorale Ecosystèmes Naturels (EDEN), University of Mahajanga, 5 Rue Georges V—Immeuble KAKAL, Mahajanga Be, B.P. 652, Mahajanga 401, Madagascar; 4 Faculté des Sciences, de Technologies et de l’Environnement, University of Mahajanga, 5 Rue Georges V—Immeuble KAKAL, Mahajanga Be, B.P. 652, Mahajanga 401, Madagascar; 5 CNRS-UPS-IRD, UMR5174, Laboratoire Évolution & Diversité Biologique, Université Paul Sabatier, 118 Route de Narbonne, 31062 Toulouse, France; 6 Department of Biology, Duke University, Durham, NC 27708, USA

The published article [1] has been retracted at the request and with the agreement of all authors.

Following publication, further data analyses showed that the hybridization signal, which forms the core of the paper, is most likely the result of cross-sample contamination.

First, the distribution of mitochondrial DNA (mtDNA) coverage among *M. murinus* samples is concerning and indicative of contamination. The authors’ previous analyses had shown that *M. murinus* mitochondrial DNA does not contain a RAD cutting site when digested with the restriction enzyme SbfI (besides very few individuals which may carry a mutation), while mtDNA of *M. ravelobensis* has a cutting site in the COI gene. Nevertheless, some samples of *M*. *murinus* show a very low mtDNA coverage and at least some (if not all) of these low mtDNA coverages were recently confirmed to be of *M. ravelobensis* origin, suggesting the presence of very low levels of contaminants (from laboratory work or barcode synthesis). This explanation is the most parsimonious one since proper hybrids should either show high coverage of the *M. ravelobensis*-type (if they are a maternal *M.* *ravelobensis*-type hybrid) or no coverage at all (if they are a maternal *M.* *murinus*-type hybrid).

The authors also recently leveraged properties of the minor allele coverage to infer the most likely ploidy of samples (i.e., identifying non-diploid individuals) and to inspect the individual profiles of previously inferred “hybrids”, using nQuire [2] and the R package ‘Vanquish’ [3]. These analyses revealed that most “hybrids” have a non-diploid signature and that, in particular, the individual with the highest levels of nuclear admixture in the manuscript shows a multimodal minor allele coverage distribution, likely originating from the mixture of two diploid samples from *M. murinus* and *M. ravelobensis* prior to RAD Sequencing, and not from hybridization. Taken together these tests leave little doubt that the original publication on hybridization between two sympatric mouse lemur species is compromised by some level of cross-sample contamination.

Consequently, and as a necessity following good scientific practice, the authors contacted the journal regarding the new findings and conclusions and asked for the retraction of the article. They apologize for any inconvenience caused by the retraction.

This retraction was approved by the Editor in Chief of the journal *Genes*.
